# Endothelial glycocalyx disruption after cardiac surgery in infants

**DOI:** 10.1186/cc9502

**Published:** 2011-03-11

**Authors:** V Sheward, S Tibby, H Bangalore, A Durward, I Murdoch

**Affiliations:** 1 Evelina Children's Hospital, London, UK

## Introduction

The endothelial glycocalyx (EGX) modulates vascular permeability and inflammation. It is disrupted by ischaemia-reperfusion. We hypothesised that cardiopulmonary bypass would elevate markers of EGX shedding, which would be associated with increased postoperative inflammation.

## Methods

A prospective cohort of 25 infants (median weight 5 kg) undergoing surgery for congenital heart disease. Blood temporal pro-files of two markers of EGX disruption - heparan sulphate (HEP) and syndecan-1 (SYND) - were correlated with a biochemical marker of systemic inflammation (IL-6) and clinical outcome variables.

## Results

**Figure 1:**
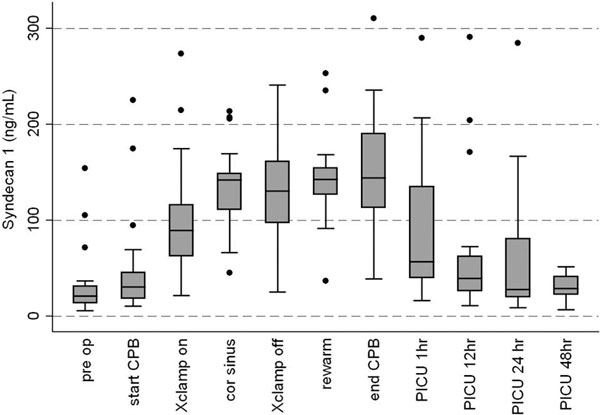
**Syndecan-1 profile**.

Infants showed a dramatic rise in SYND, which peaked at the end of bypass, returning to baseline at 48 hours (Figure 1). The median (IQR) peak SYND levels were 144 ng/ml (113 to 190), representing a sixfold rise from baseline. A less pronounced rise was seen for HEP (median 22.5 μg/ml), which approximately doubled. Peak IL-6 occurred at 12 hours post bypass: median 118 pg/ml (44 to 217). Absolute peak values of both SYND and HEP correlated poorly with IL-6 and all clinical variables. Conversely, peak IL-6 correlated with bypass time (*r *= 0.53), length of ventilation (*r *= 0.69) and ICU stay (*r *= 0.58).

## Conclusions

Although markers of EGX disruption show a reproducible temporal profile after bypass, the lack of correlation with IL-6 and clinical markers means that their significance is unclear.

